# Goat milk extracellular vesicles: immuno-modulation effects on porcine monocyte-derived macrophages *in vitro*

**DOI:** 10.3389/fimmu.2023.1209898

**Published:** 2023-06-16

**Authors:** Giulia Franzoni, Samanta Mecocci, Chiara Grazia De Ciucis, Lorena Mura, Filippo Dell’Anno, Susanna Zinellu, Floriana Fruscione, Livia De Paolis, Tania Carta, Antonio G. Anfossi, Silvia Dei Guidici, Elisabetta Chiaradia, Luisa Pascucci, Annalisa Oggiano, Katia Cappelli, Elisabetta Razzuoli

**Affiliations:** ^1^Department of Animal Health, Istituto Zooprofilattico Sperimentale della Sardegna, Sassari, Italy; ^2^Department of Veterinary Medicine, University of Perugia, Perugia, Italy; ^3^National Reference Center of Veterinary and Comparative Oncology (CEROVEC), Istituto Zooprofilattico Sperimentale del Piemonte, Liguria e Valle d’Aosta, Genova, Italy; ^4^Department of Biomedical Sciences, School of Medicine, University of Sassari, Sassari, Italy; ^5^Department of Veterinary Medicine, University of Sassari, Sassari, Italy

**Keywords:** milk extracellular vesicles, pig, macrophages, classical activation, cytokines, toll-like receptors

## Abstract

**Introduction:**

Extracellular vesicles (EVs) are nanometric-membrane-bound sub-cellular structures, which can be recovered from milk. Milk EVs have drawn increasing interest due to their potential biomedical applications, therefore it is important to investigate their impact on key immune cells, such as macrophages.

**Methods:**

In this work, the immunomodulatory effects of goat milk EVs on untreated (moMФ) and classically activated (moM1) porcine monocyte-derived macrophages were investigated using flow cytometry, ELISA, and gene expression assays.

**Results:**

These particles were efficiently internalized by macrophages and high doses (60 mg protein weight) triggered the upregulation of MHC I and MHC II DR on moMФ, but not on moM1. In moMФ, exposure to low doses (0.6 mg) of mEVs enhanced the gene expression of IL10, EBI3, and IFNB, whereas high doses up-regulated several pro-inflammatory cytokines. These nanosized structures slightly modulated cytokine gene expression on moM1. Accordingly, the cytokine (protein) contents in culture supernatants of moMФ were mildly affected by exposure to low doses of mEVs, whereas high doses promoted the increased release of TNF, IL-8, IL-1a, IL-1b, IL-1Ra, IL-6, IL-10, and IL-12. The cytokines content in moM1 supernatants was not critically affected.

**Discussion:**

Overall, our data support a clinical application of these molecules: they polarized macrophages toward an M1-like phenotype, but this activation seemed to be controlled, to prevent potentially pathological over-reaction to stressors.

## 1 Introduction

Extracellular vesicles (EVs) are micrometric and nanometric sub-cellular structures, enclosed in a lipid bilayer membrane and secreted by multiple cell types under specific physiological and pathological conditions. EVs mediate the intercellular cross-talk between the producing and the target cell, through the transfer of a cargo containing different types of molecules (lipids, proteins, metabolites, and nucleic acids) which display immunomodulatory properties. Being released in the extracellular environment, EVs can be recovered from any biological fluids, including milk (1). Milk is among the most promising sources of EVs, since it allows a considerable recovery of EVs (mEVs). This is a pivotal point, since mEVs have a great theranostic potential that can be exploited for different biomedical applications (1). Regardless of their origin, EVs may be used for diagnostic purposes carrying potential biomarkers of pathological conditions or for monitoring a therapy response, but can also be utilized as therapeutic agents themselves or as drug delivery systems (2, 3). The latter two applications are particularly appropriate for mEVs thanks to their massive production and the intrinsic biological functions related to the natural cargo (1, 4). Beneficial effects exerted by mEVs and their molecular cargo can occur at multiple levels, being able to modulate cellular processes related to immunity, inflammation, cell homeostasis, bone and muscle metabolism, organism growth and development, and the microbiota composition (5). In addition to these properties, several types of molecules can be loaded into mEVs, like anti-cancer drugs, small RNAs, and anti-inflammatory and antioxidant agents, although this field of research is in its infancy (6). Several methods have been studied to load drugs into EVs, as well as evaluation of donor cells, and attachment of targeting molecules (6).

In view of their biomedical application, it is crucial to investigate the interaction of these nanosized structures with the immune system, since any exposure to foreign materials will inevitably lead to an encounter with the immune cells, in particular with phagocytic cells, like macrophages. Macrophages are cells of the innate immune system, involved in a broad array of functions, spanning from tissue homeostasis to immune responses against invading pathogens (7). They are professional phagocytes, which can quickly respond to endogenous danger signals generated following injuries or infections. They recognize pathogen-associated molecular patterns (PAMPs) and danger-associated molecular patterns (DAMPs) through several pattern-recognition receptors (PRRs), such as toll-like receptors (TLRs) (8). They are involved in defense against both infective and not infective stressors (7). The impact of foreign molecules on these professional phagocytes may indeed benefit treatment or trigger unwanted immunotoxicity. Macrophages have extraordinary plasticity, as they can change their phenotype and functions in response to environmental signals (9). An extreme of macrophage polarized status is represented by classically activated macrophages (M1), which can be generated *in vitro* using IFN-γ and lipopolysaccharide (LPS). This subset presents a pro-inflammatory phenotype, and its primary role is in host defense to intracellular pathogens and in driving Th1 cellular immune responses (9). Their activation must be tightly controlled, in order to avoid unwanted exacerbated reactions (9). In addition, macrophages have become an important therapeutic target for the treatment of diverse diseases, such as rheumatoid arthritis, atherosclerosis, and cancer (10). In this context, we investigated the impact of goat mEVs on both untreated (moMФ) and classically activated (moM1) macrophages.

In this *in vitro* study, we used porcine monocyte derived macrophages. Pigs were chosen as they represent a close to human model, broadly used in translational studies spanning from preclinical evaluation of therapeutics and vaccine candidates (11, 12) as well as preclinical toxicologic testing (13, 14). It has been also suggested that pigs are a better model than rodents to understand human innate immunity (15), and it was reported that porcine macrophages resembled human macrophages after stimulation with a TLR4 ligand (LPS), responding with an analogous inducible gene expression profile (16, 17). In addition, porcine moM1 are broadly comparable to human moM1, being characterized by enhanced expression/release of pro-inflammatory cytokines, up-regulated expression of MHC molecules (MHC class I and II), and co-stimulatory molecules (18, 19).

Overall, in this study the anti-inflammatory and immunomodulating effects of goat mEVs on porcine macrophage subsets were investigated in detail, using a vast array of techniques spanning from flow cytometry, microscopy, multiplex and singleplex ELISA, and gene expression assays.

## 2 Material and methods

### 2.1 Milk sampling

A local farm in Perugia (Italy) was chosen for milk sampling. The farm is routinely monitored and surveilled by the Department of Veterinary Medicine of Perugia University. In order to reduce problems related to interindividual variability, bulk tank goat milk was collected and immediately processed or stored at 4°C for less than 24h, avoiding any intermediate cryo-preservation in order to reduce artifacts.

### 2.2 Extracellular vescicles (isolation and characterization

To isolate mEVs the protocol tuned for milk by Mecocci and collaborators (20) was applied. Briefly, fresh milk was subjected to two preliminary centrifugations for 10 minutes at 3000 x g at RT in an Eppendorf^®^ Centrifuge 5810R with an F34‐6‐38 rotor. This passage allows the elimination, from raw milk, of fat globules and cells/cell debris in the upper layer and in the pellet, respectively. Then ethylenediaminetetraacetic acid tetrasodium salt dihydrate (EDTA, 0.25 M, pH 7.4) was added to the supernatant in a 1:1 ratio, left in ice for 15 minutes, and centrifuged for 1 h at 4°C at 10000 x g. To eliminate as much of the protein aggregates as possible, the recovered supernatant was further centrifuged for 1 h at 4°C at 35000 x g (Beckman Coulter Optima L‐100 XP with a 45 Ti rotor). At last, the supernatant was collected and a final ultracentrifugation for 90 minutes at 4°C at 200000 x g was used. Pellets containing mEVs were resuspended in sterile filtered (0.22 μm) phosphate-buffered saline (PBS, 1 X) (Euroclone, Pero, Italy) and stored at -80°C until use (20).

Isolated mEVs were then morphologically characterized with Transmission Electron Microscopy (TEM) and Western Blotting (WB). WB was employed to test the presence of EV marker proteins. Briefly, mEVs were lysed in RIPA Buffer containing 25 mM Tris-HCl pH 7.6, 150 mM NaCl, 1% NP-40, 1% sodium deoxycholate, 0.1% SDS. After quantification of protein concentration by using Bradford assay, 25 μg of total proteins were resolved in 12% sodium dodecyl sulfate–polyacrylamide gel electrophoresis and transferred on nitrocellulose membranes. Blotted membranes were saturated in 0.5% of bovine serum albumin, and then incubated O.N. at 4°C with the primary antibodies against CD81 (1:500; Bioss Antibodies, Woburn, MA, USA), TSG-101 (1:400, Santa Cruz Biotechnology, Santa Cruz, CA, USA) and calnexin (1:400 sc-23954, Santa Cruz Biotechnology, Santa Cruz, CA, USA). After 2 washing steps, the appropriate secondary antibody, i.e. anti-rabbit HRP-conjugated IgG (1:3000, Cell Signaling Technology, Danvers, MA, USA) and anti-mouse HRP-conjugated IgG (1:3000, Cell Signaling Technology), was added and incubation was carried out for 1 hour at room temperature. Immunoreactivities were highlighted by using Clarity Western ECL Substrate (Bio-Rad). The film images were acquired by using a GS-800 imaging systems scanner (Bio-Rad, Hercules, CA, USA). A small aliquot (10 μl) of mEVs suspension was put on Parafilm for TEM analysis. mEVs were allowed to stick to formvar-coated copper grids (Electron Microscopy Sciences) for 20 minutes with the coated side facing the suspension. After being rinsed in PBS and distilled water, grids were contrasted for 5 minutes with 2% uranyl acetate. Philips EM208 transmission electron microscope with a digital camera (University Centre of Electron and Fluorescence Microscopy—CUMEF) was used for observation. The same preparations were also tested for concentration and size distribution through nanoparticle tracking analysis (NTA) and results were already reported in Mecocci et al. (21).

### 2.3 Blood donor pigs and ethical statement

Five cross-bred pigs (Sus scrofa domesticus), either male or female, 6–18 months old, were used to donate blood for macrophage generation. Animals were kept at the Experimental Station of Istituto Zooprofilattico Sperimentale (IZS) of Sardinia (Sassari, Italy). Animal husbandry, handling, and procedures (bleeding) were performed in accordance to the Italian Legislative Decree n.26 dated 4th of March 2014, as well as the Guide of Use of Laboratory Animals issued by the Italian Ministry of Health (authorization n° 1232/2020-PR). Animal health status was controlled by authorized veterinarians, and samples (EDTA blood) were routinely screened for main porcine pathogens, as previously described (22). In detail, qualitative real-time PCR was employed to exclude the presence of porcine parvovirus (PPV), porcine circovirus 2 (PCV2), and African swine fever (ASFV) genome (22, 23), with primers reported in the **Table S1** (24–26), whereas commercial real-time PCR kits were used to detect porcine reproductive and respiratory syndrome virus (PRRSV), and Mycoplasma hyopneumoniae genome (LSI VetMAX™ PRRSV EU/NA and VetMAX™-Plus qPCR Master Mix, both Thermo Fisher Scientific, respectively) (22).

### 2.4 Production of porcine monocyte-derived macrophages, differentiation, and stimulation with diverse doses of EVs

MoMФ cultures were obtained from heparinized blood samples, as we previously published (22). In brief, leukocytes were cultured in RPMI-1640 supplemented with 10% foetal bovine serum (FBS), antibiotics (100 U/mL penicillin, and 100 μg/mL streptomycin) (complete RPMI, cRPMI), and recombinant human M-CSF (hM-CSF) (Thermo Fisher Scientific, Waltham, MA, USA) (final concentration 50 ng/mL), using Petri dishes (22, 27). Cells were then seeded in 12-well plates (Greiner CELLSTAR, Sigma-Aldrich, Saint Louis, MO, USA) (1 × 10^6^ live moMФ per well) or 4-well chamber slides (Nunc Lab-Tek chamber slide system, Thermo Fisher Scientific) (3 × 10^5^ live moMФ per well). After seeding, macrophages were cultured in un-supplemented fresh cRPMI at 37°C 5% CO_2_. 24 h later, moMФ were left untreated or differentiated into moM1, using recombinant porcine IFN-γ (Raybiotech Inc, Norcross, GA, USA) and LPS (lipopolysaccharide from Escherichia coli 0111:B4; Sigma-Aldrich), both at a concentration of 100 ng/mL (27). 24 h later, both moMФ and moM1 cultures were left untreated or exposed to diverse doses of goat mEVs (0.6, 60 μg) for 24 or 48h. In selected experiments (see 2.5), moMФ were instead exposed to scalar doses of goat milk EVs for 24h: 0.06, 0.6, 6, 60, 600 μg (protein weight).

### 2.5 Cell viability

MoMФ were seeded in 12-well plates and 24h later were exposed to scalar doses of goat milk EVs (0.06, 0.6, 6, 60, 600 μg), alongside untreated control. After 24 h, cell viability was determined using Cytotox 96^®^ Non-Radioactive Cytotoxicity Assay (Promega, Madison, WI, USA), according to manufacturer’s instructions. In detail, the amounts of lactate dehydrogenase (LDH) in culture supernatants were quantified using this non-radioactive cytotoxicity assay, using a lysis solution provided by the manufacturer as a positive control. Absorbance was read at 492nm using an Epoch microplate reader (BioTek, Winoosky, USA) (28).

### 2.6 Internalization assay

MoMФ were seeded in 4-well chamber slides and were exposed to goat milk EVs, alongside control. Before addition to the cell monolayer, EVs were labeled using the PKH67 Green Fluorescent Cell Linker Kit for General Cell Membrane Labeling (Sigma-Aldrich), following the manufacturer’s direction. In brief, 60 μg (protein weight) EVs were resuspended in 0.5 mL dye buffer, then 2 μL of the green fluorescent dye PKH67 was diluted in 0.5 mL dye buffer and finally these were added and mixed. As a control, PBS without EVs was used: 0.2 mL of PBS was added to 0.5 mL dye buffer and 2 μL of the green fluorescent dye PKH67 and processed in parallel to EVs samples. After 5 min of incubation, 0.5 mL of PBS containing 1% bovine serum albumin (BSA, Sigma-Aldrich) was added to stop the labeling reaction. Labeled EVs were washed by ultracentrifugation at 100,000 x g for 1 h, diluted in a complete culture medium, and added to macrophages (29). 24 h post-treatment, macrophages were fixed with 4% paraformaldehyde, washed with PBS, and fluorescence microscopy images were acquired using a fluorescent microscope (Olympus IX70, Segrate, Italy) equipped with a 40 X/0.40 numeric aperture objective lens (22).

### 2.7 Flow cytometry

MoMФ or moM1 were seeded in 12 well plates and treated with EVs, alongside untreated control. 24 and 48 h post-stimulation, flow cytometry was carried on to determine the surface expression of MHC I and MHC II DR, as well as dimension (forward scatter area, FSC-A, geometric mean), as previously described (22, 30). In brief, cells were harvested with 10 mM EDTA in PBS, transferred to 5 mL round bottom tubes (Corning), and stained with Zombie Aqua viability dye (BioLegend, San Diego, CA, USA). After incubation (30 min, RT), cells were stained with anti-pig MHC I (clone JM1E3, Bio-Rad Antibodies), and anti-pig MHC II DR (clone 2E9/13, Bio-Rad Antibodies). Expressions of these molecules were then visualized by subsequent staining with BV421 rat anti-mouse IgG1 (clone A85-1, BD Horizon BD Biosciences, Franklin Lakes, NJ, USA) or BV786 rat anti-mouse IgG2b (clone R12-3, BD Horizon BD Biosciences), respectively. Analyses were performed using a FACS Celesta flow cytometer (BD Biosciences) and BD FACS Diva Software (BD Biosciences). 5000 live macrophages were acquired, then data analyses were carried out by exclusion of doublets, gating on viable cells, with subsequent assessment of MHC molecules (MHC I or MHC II DR) staining (**Figure S1**) (22, 30).

### 2.8 Cytokine release in response to EVs treatment

At 24 and 48h post-treatment, culture supernatants were collected and cellular debris was removed by centrifugation (at 2500×g for 3 min). Supernatants were then stored at −80°C until analyzed. Levels of GM-CSF, TNF, IL-1α, IL-1β, IL-1Ra, IL-6, IL-8, IL-10, IL-12, and IL-18 were determined using the Porcine Cytokine/Chemokine Magnetic Bead Panel Multiplex assay (Merck Millipore, Darmstadt, Germany) and a Bioplex MAGPIX Multiplex Reader (Bio-Rad, Hercules, CA, USA), following manufacturer’s instructions (21). The amount of IFN-β was instead quantified using a sandwich enzyme immunoassay (porcine IFN-β ELISA kit, MyBiosource, San Diego, CA, USA), following the manufacturer’s directions, reading absorbance at 450nm with an Epoch microplate reader (BioTek) (28).

### 2.9 Impact of diverse polarizing factors on key immune gene expression

Twelve (12) well plates were used to seed MoMФ or MoM1, left untreated or stimulated with diverse EV quantities (0.6 or 60 μg) (as described in Section 2.4). After 0, 24, and 48 h, the gene expression of selected key immune genes was evaluated in harvested cells. Total RNA was isolated with an RNeasy Mini Kit (Qiagen s.r.l., Milan, Italy) and Qiacube System (automated nucleic acid extraction system - Qiagen s.r.l., Milan, Italy), and a quali-quantitative evaluation was performed using Qubit 3.0 Fluorometer (Thermo Fisher Scientific, Waltham, MA, USA). An amount of 250 ng for each RNA sample was used as template for reverse transcription with iScript^®^ cDNA Syntesis Kit (Bio-Rad, Milan, Italy). RT-qPCR was assessed, on CFX96™ System (Bio-Rad, Milan, Italy) to evaluate the expression of several key immune genes, as previously described (21, 22). Genes of interest are interleukins (IL) *IL1B*, *IL6*, *IL10*, *IL12A*, *IL12B*, and *IL18* and IL-8 gene *CXCL8*; toll-like receptors *TLR1*, *TLR2*, *TLR3*, *TLR4*, *TLR5*, *TLR7*, *TLR8*, and *TLR9*; beta-defensin 1 (*DEFB1*), interferon-beta (*IFNB*), tumor necrosis factor alfa (*TNFA*), Epstein-Barr virus induced 3 (*EBI3*), and nuclear factor kappa-light-chain-enhancer of activated B cells subunit p65 (*RELA*). Primer sequences of targets and reference genes (glyceraldehyde 3-phosphate dehydrogenase (GAPDH) and the corresponding reference, are reported in **Table S2** (31–35). Five independent experiments using different blood donor pigs were performed for all genes monitored. The relative gene expression levels were calculated from Cq (quantification cycle) values, using the 2^-ΔΔCq^ method (36), as we previously carried out (21, 22, 27).

### 2.10 Statistical analysis

Experiments were carried out in technical duplicate (flow cytometry, RT-qPCR, ELISA) or triplicate (cytotoxic assay), using at least three different blood donor animals (biological replicates). Data were analyzed using GraphPad Prism 9.01 (GraphPad Software Inc., La Jolla, CA, USA): first, data were checked for normality using the Shapiro–Wilk test, then they were graphically and statistically analyzed. Data were presented as mean and standard deviation and were analyzed using either the Student’s unpaired t-test or the non-parametric Mann-Whitney U test. The significance threshold was set at p<0.05.

## 3 Results

### 3.1 Milk EVs characterization and selection of concentrations for *in vitro* experiments

**Figure 1:**
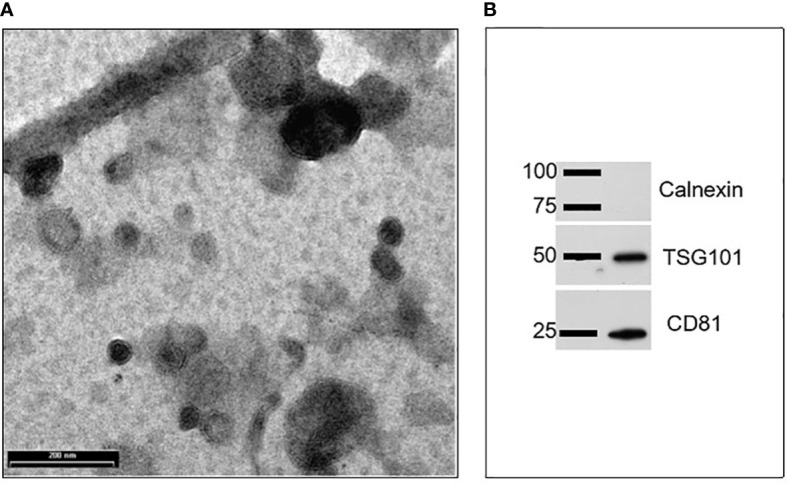
Characterization of goat milk EVs: **(A)** Transmission electron microscopy (TEM) revealed the presence of vesicles mainly in the range of 30–200 nm. Scale bar: 200 nm; **(B)** Representative images of western blots performed to evidence the presence of positive EV protein markers (such as Tsg101 and CD81) and the absence of a negative EV protein marker calnexin.

EVs were isolated from goat milk following the procedure described in our previous studies (20). The presence and purity of EVs in the pellet, as well as their size range and shape, were evaluated through Transmission Electron Microscopy (TEM) and Western blotting (WB). As shown in **Figure 1**, TEM analysis revealed the presence of EVs, which were homogeneous in shape with intact limiting membrane. Their size varied between 30 and 500 nm, with most of them not exceeding a diameter of 200 nm. They were sometimes aggregated and showed a variable electron density (**Figure 1A**). Wester blotting analysis confirmed the presence of positive EV marker proteins such as tumor susceptibility gene 101 (Tsg101) and a cluster of differentiation 81 (CD81), but not for calnexin, the latter regarded as negative EV marker protein (**Figure 1B**).

**Figure 2:**
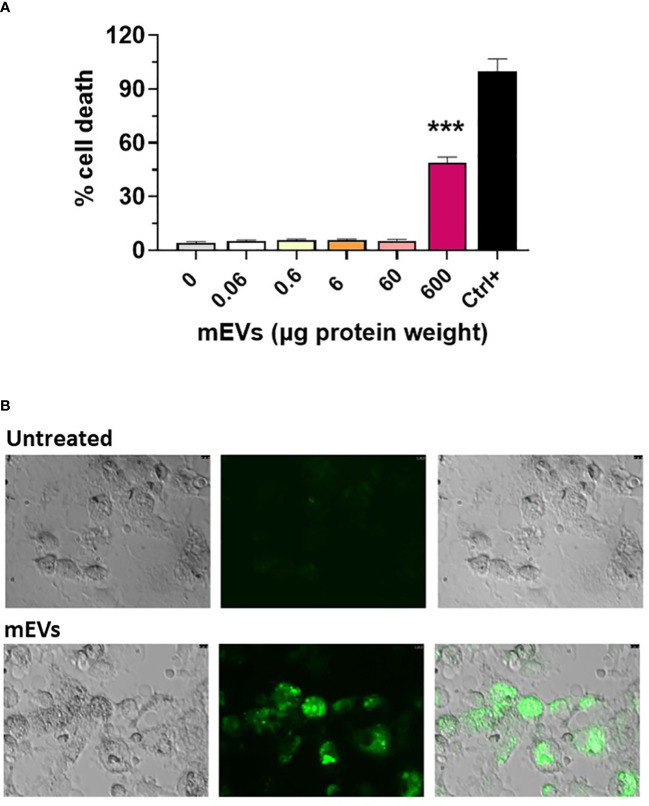
Goat mEVs impact on moMФ cell viability and internalization. **(A)** Porcine moMФ were left untreated (0) or treated with scalar doses of goat mEVs: 0.06, 0.6, 6, 60, 600 μg protein weight. After 24h, viability was assessed using a non-radioactive cytotoxic assay, with a lysis solution as positive control (‘Ctrl+’). Mean data and SD from three independent experiments are displayed; values for mEVs-treated samples were compared to untreated control using an unpaired Mann-Whitney test; ***p < 0.001. **(B)** Goat mEVs were labeled with the green fluorescent dye PKH67 and then were incubated with porcine moMФ, alongside corresponding untreated condition. After 24h, cells were morphologically evaluated through fluorescent microscopy. Images of representative moMФ, either untreated or exposed to mEVs are presented. Scale bar, 7.5 µm.

Goat milk EVs’ impact on porcine moMФ viability was assessed through a non-radiolabeled immunoassay. Cells were exposed to scalar doses of these particles (0, 0.06, 0.6, 6, 60, 600 μg protein weight), and 24h later LDH amounts in culture supernatants were measured using a cytotoxicity non-radioactive assay. Cell viability decreased only when goat mEVs were added at the highest dose: 600 μg (**Figure 2A**). Accordingly, we selected two amounts of goat mEVs to investigate the immunomodulatory properties of these particles on porcine macrophages: 0.6 and 60 μg. Then, the ability of these cells to efficiently internalize goat mEVs was assessed by microscopy and, as expected, these molecules were taken up effectively by porcine macrophages (**Figure 2B**).

The impact of goat mEVs on both porcine untreated (moMФ) and classically activated macrophages (moM1) was analyzed in this study. Their immunomodulatory effects were investigated through flow cytometry, gene expression, and multiplex ELISA. Classical activation was confirmed by the upregulation of activation markers (MHC I and MHC II DR) (**Figure S2**), and the induction/release of pro-inflammatory cytokines (**Figures S3**, **S4**).

### 3.2 Milk EVs impact on MHC I and MHC II DR expression on porcine moMФ and moM1

**Figure 3:**
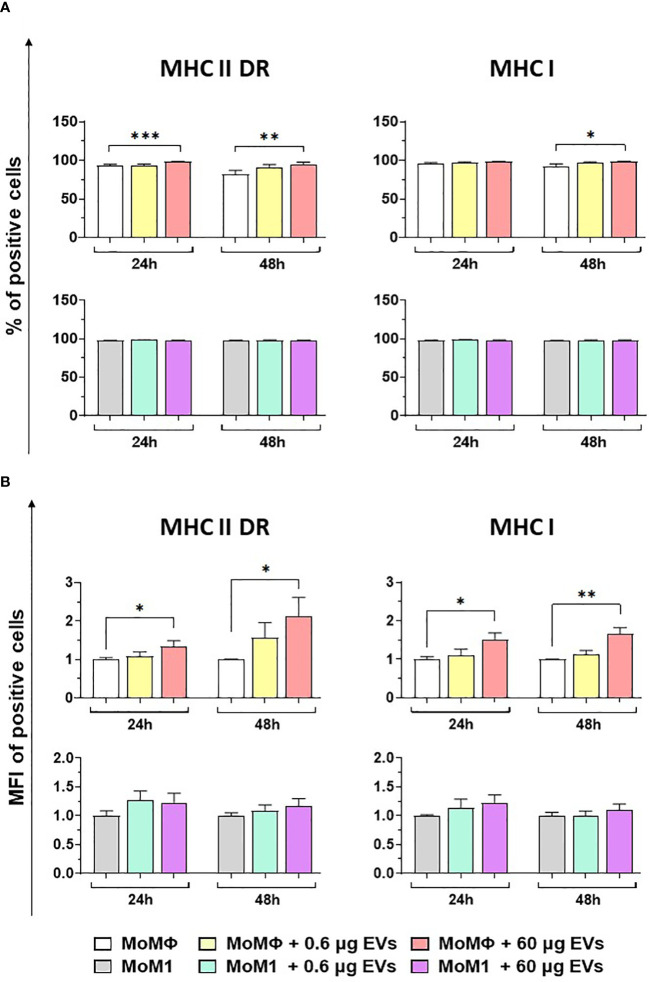
Effect of goat mEVs on MHC I and MHC II DR surface marker expressions. Porcine moMФ or moM1 were left untreated or treated with 0.6 or 60 μg of goat mEVs. 24 and 48 h post-stimulation, flow cytometry was employed to determine surface expression of MHC I and MHC II DR, either in terms of percentage of positive cells **(A)** or mean fluorescent intensity (MFI) of positive cells **(B)**. MFI data are expressed as fold change relative to the untreated condition. Mean data and SD from four independent experiments are displayed. Values of treated macrophages were compared to the untreated control (moMΦ or moM1), using an unpaired T test of a Mann-Whitney test; ***p < 0.001, **p < 0.01, *p < 0.05.

Flow cytometry was employed to quantify the expression of MHC molecules (MHC class I and II DR) on macrophage subsets 24 and 48 h after exposure to mEVs (**Figure S1**). Our data revealed that the treatment with 60 μg of goat mEVs, but not 0.6 μg, triggered the upregulation of both MHC II DR and MHC I on moMФ, both appreciated in terms of percentages of positive cells and mean fluorescence intensity (MFI) of positive cells (**Figure 3**). As expected, classical activation (IFN-γ + LPS) triggered the upregulation of these surface markers (**Figure S2**); we observed that in this subset exposure to the tested particles (high or low doses) did not result in modulation of either MHC I or MHC II DR at any tested time points (24 and 48 h post-treatment) (**Figure 3**). Flow cytometry revealed also a small but statistical significant increase in the size of macrophage subsets after exposure to goat mEVs, mainly appreciated in moMФ treated with high doses of these nanoparticles (**Figure S5**).

### 3.3 Milk EVs modulation of key immune genes in porcine moMФ and moM1

**Figure 4:**
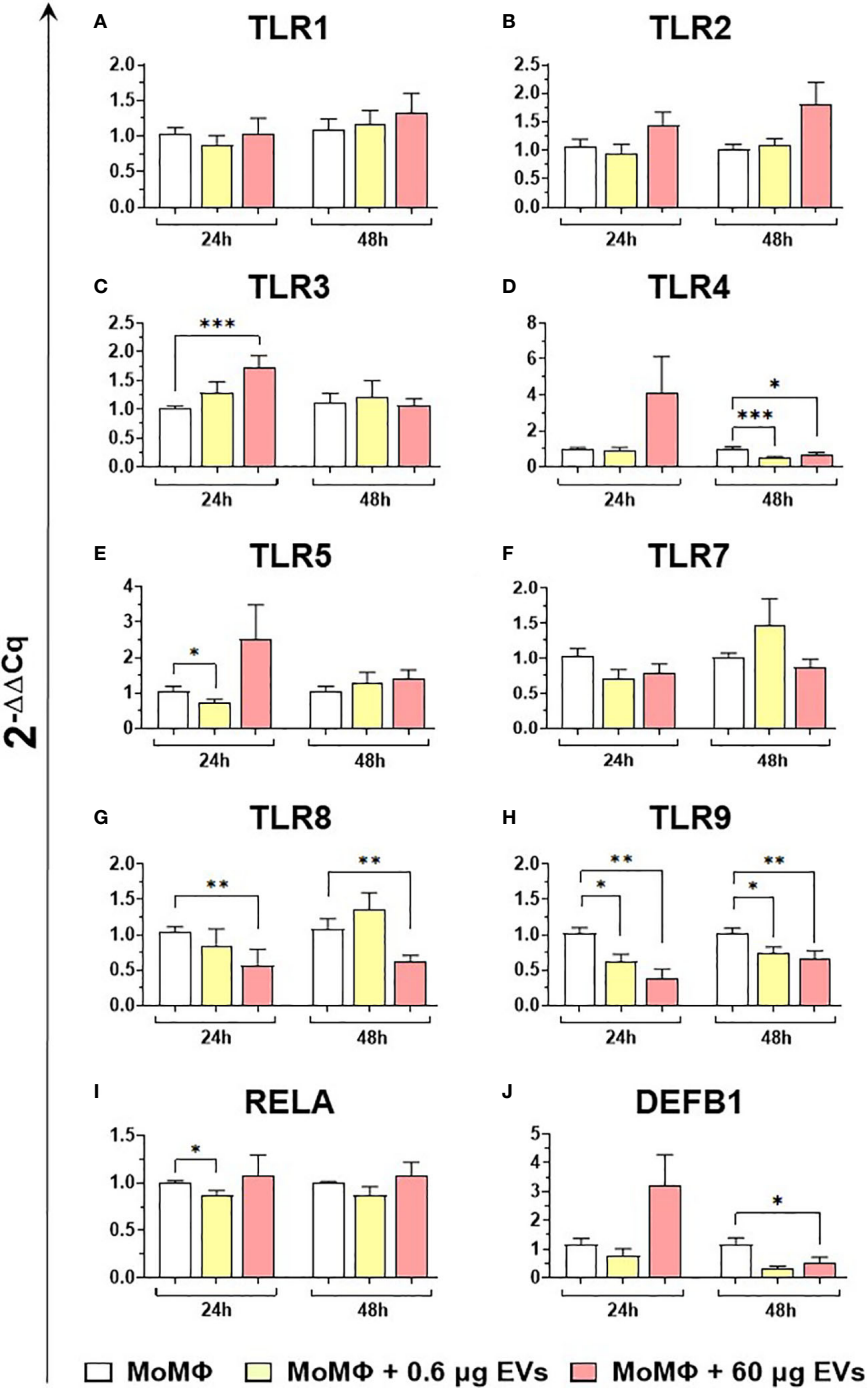
moMФ expression of innate immunity related genes after treatment with diverse doses of goat mEVs. Porcine moMФ were left untreated or treated with 0.6 or 60 μg mEVs for 24 and 48 h. Gene expression levels of the eight TLRs **(A–H)**, the the NFKB-p65 subunit (RELA) **(I)**, and beta-defensin 1 (DEFB1) **(J)** were determined using RT-qPCR. Data derived from cells after exposure were normalized on the values of reference gene expression and untreated control group (moMΦ) and expressed as 2^−ΔΔCq^. Mean data and SD from five independent experiments using different blood donor pigs are displayed. Values of treated macrophages were compared to the untreated control (moMΦ), using the unpaired t-test or the Mann-Whitney U test; ***p < 0.001, **p < 0.01, *p < 0.05.

**Figure 5:**
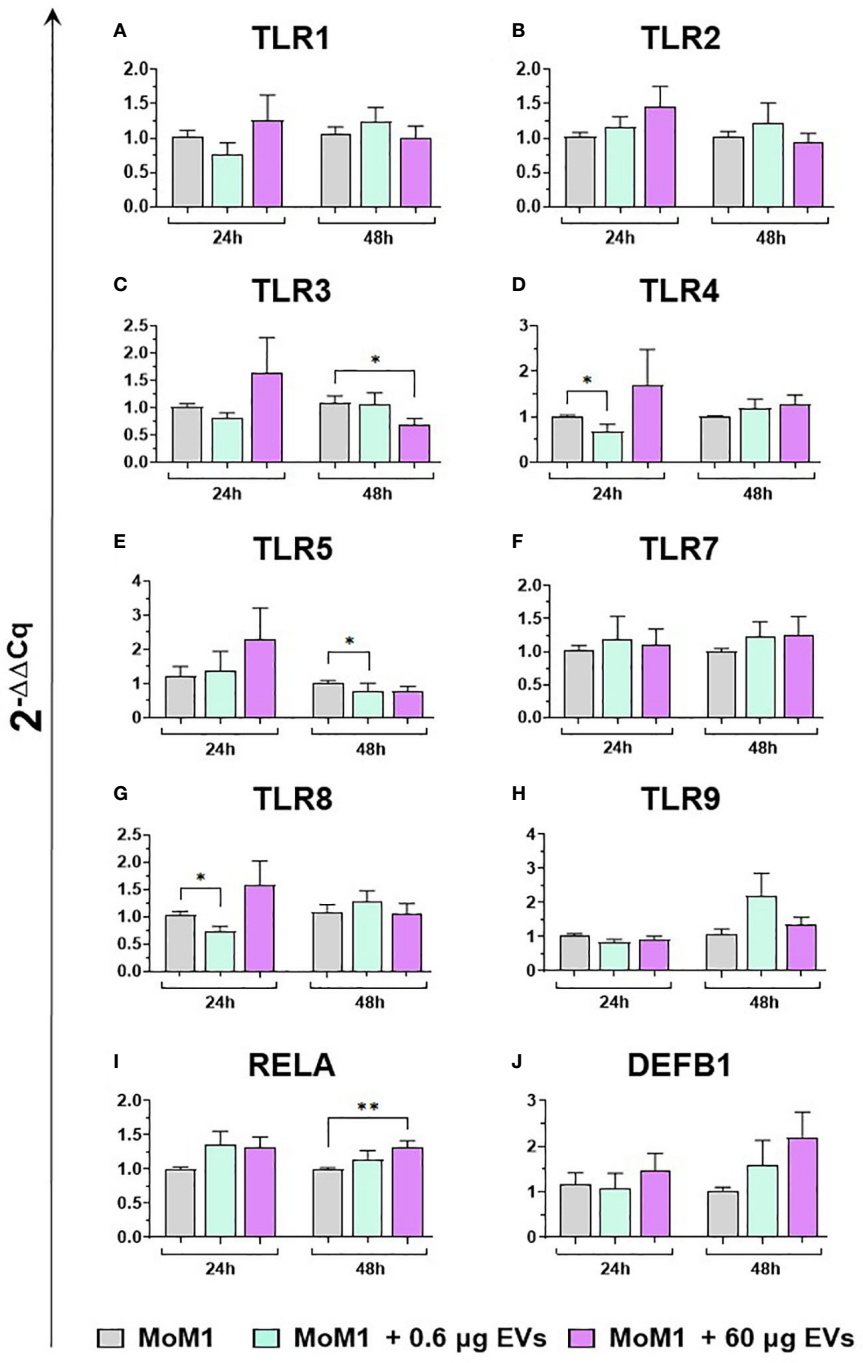
moM1 expression of innate immunity related genes after treatment with diverse doses of goat mEVs. Porcine moM1 were left untreated or treated with 0.6 or 60 μg mEVs for 24 and 48 h. Gene expression modulation of eight TLRs **(A–H)**, the the NFKB-p65 subunit (RELA) **(I)**, and beta-defensin 1 (DEFB1) **(J)** was determined using RT-qPCR and the 2^−ΔΔCq^ values are reported as mean and SD from five independent experiments using different blood donor pigs. Values of treated macrophages were compared to the untreated control (moMΦ), using an unpaired t-test or a Mann-Whitney U test; **p < 0.01, *p < 0.05.

We subsequently investigated the impact of the two doses of goat mEVs on the gene expression of key immune molecules on macrophage subsets, including toll-like receptors (TLRs). TLRs are a group of PRRs, that recognize molecules expressed by pathogens (pathogen associated molecular patterns; PAMPs) and play a central role in initiating the host immune defense (37). Eight TLRs were tested in this work, either intracellular (*TLR3*, 7, 8, and 9) or extracellular (*TLR1*, 2, 4, and 6) (37). Two other key immune genes were tested: *RELA*, and *DEF1B*. *RELA*, also known as p65, is involved in heterodimer formation, nuclear translocation, and activation of the transcription factor NF-κB, involved in TLR signaling (38). *DEF1B* encodes for beta defensin 1 (BD1), which is a host antimicrobial peptide with antimicrobial activity against a broad range of bacteria (39). We observed that low doses of goat mEVs (0.6 μg) only slightly modulated the expression of these genes by moMФ, with reduced expression of *TLR4* (48h), *TLR5* (24h), *TLR9* (both at 24h and 48h) and *RELA* (24h). On the contrary, high doses of mEVs (60 μg) enhanced the expression of *TLR3* (24h), but down-regulated the expression of *DEFB1* (48h), and three TLRs: *TLR4* (48h), *TLR8* (24 and 48h), *TLR9* (both at 24h and 48h) (**Figure 4**). The effect of mEVs was less marked on moM1: 0.6 μg reduced the expression of *TLR4* (48h) and *TLR5* (48h), while higher doses (60 μg) down-regulated *TLR3* expression (48h) and slightly up-regulated *RELA* expression (48h) (**Figure 5**).

**Figure 6:**
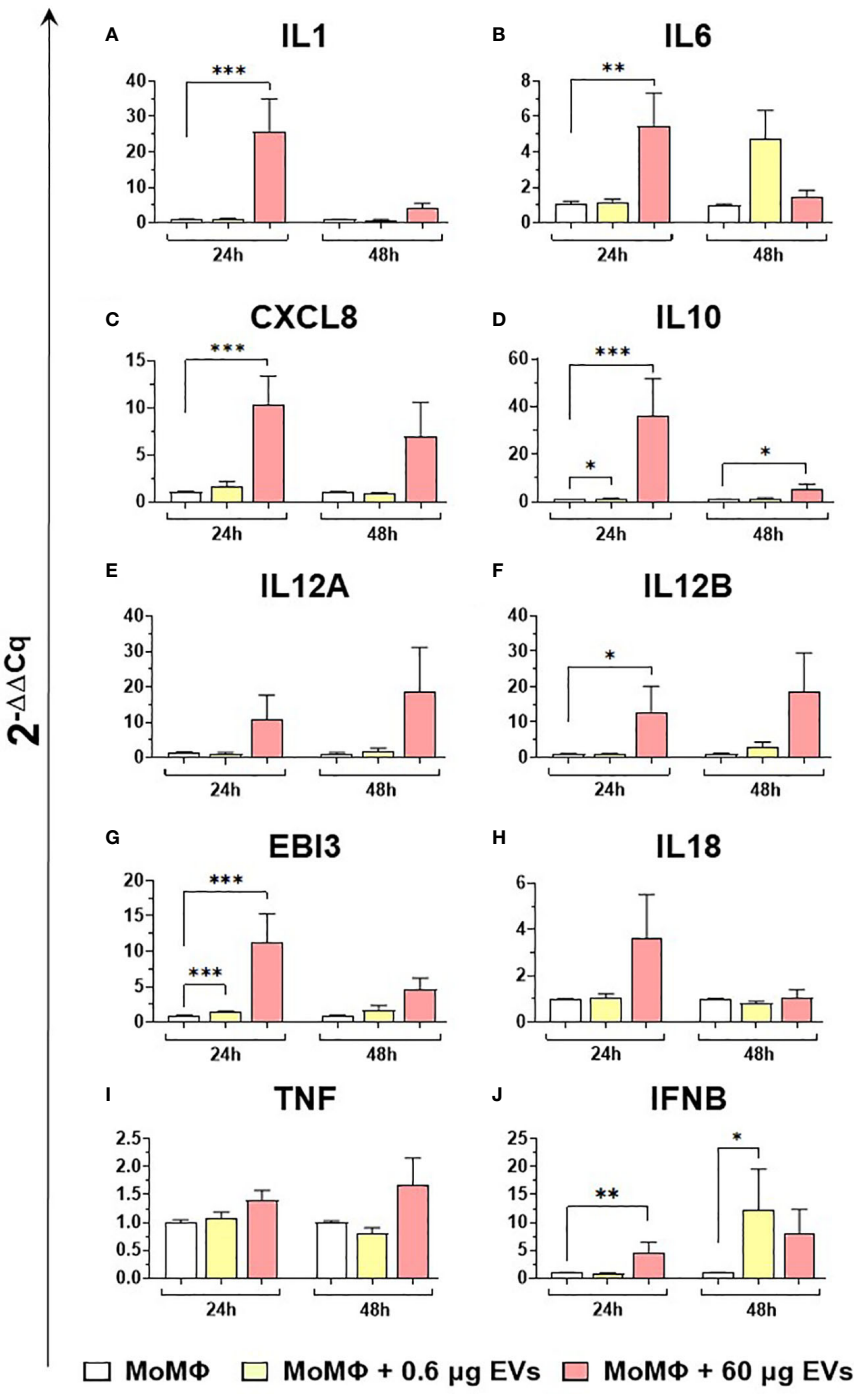
moMФ expression of cytokine genes after exposure to diverse doses of goat mEVs. Porcine moMФ were left untreated or treated with 0.6 or 60 μg mEVs for 24 and 48 h. Gene expression levels of ten cytokines (*IL1, IL6, CXCL8, IL10, IL12A, IL12B, EBI3, IL18, TNF, IFNB*) **(A–J)** were determined using RT-qPCR. At each time post-exposure, data were normalized on the values of untreated control group (moMΦ) and expressed as 2^−ΔΔCq^. Mean data and SD from five independent experiments using different blood donor pigs are displayed. Values of treated macrophages were compared to the untreated control (moMΦ), using an unpaired T test of a Mann-Whitney test; ***p < 0.001, **p < 0.01, *p < 0.05.

**Figure 7:**
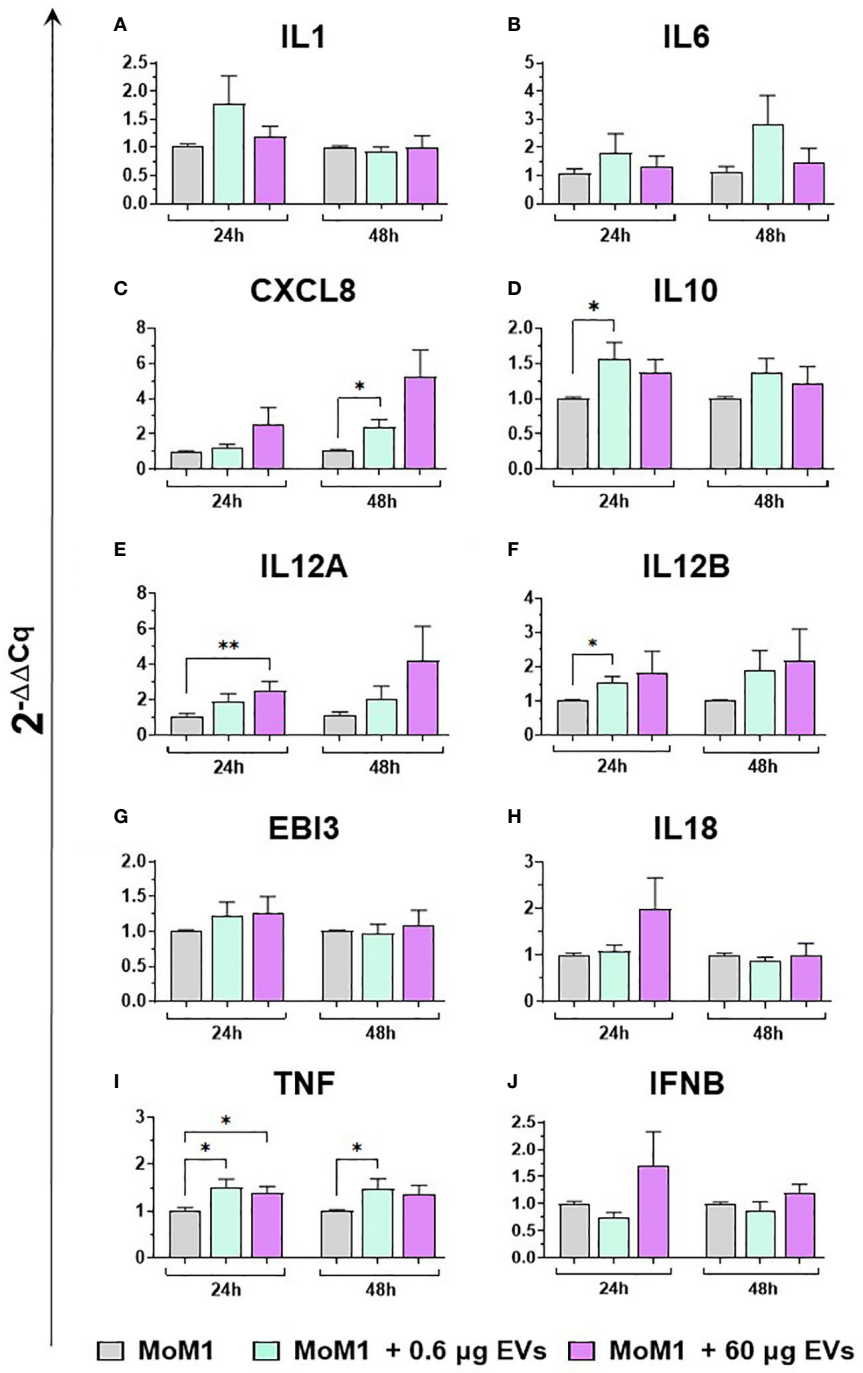
moM1 expression of cytokine genes exposed to diverse doses of goat mEVs. Porcine moM1 were left untreated or treated with 0.6 or 60 μg mEVs. At 24 and 48 h post-stimulation, gene expression levels of ten cytokines (*IL1, IL6, CXCL8, IL10, IL12A, IL12B, EBI3, IL18, TNF, IFNB*) **(A-J)** were determined using RT-qPCR. At each time post-exposure, data were normalized on the values of untreated control group (moM1) and expressed as 2^−ΔΔCq^, reporting in the graph mean values and SD of five independent experiments using different blood donor pigs are displayed. Values of treated macrophages were compared to the untreated control (moMΦ), using an unpaired T test of a Mann-Whitney test; *p < 0.05; **p < 0.01.

Then, the effects of different concentration of mEVs on the expression of ten major cytokines on macrophage subsets were analyzed. We observed that mEVs enhanced the expression of several cytokines on moMФ, although with remarkable differences between concentrations. In detail, exposure to 0.6 μg of mEVs resulted in enhanced expression of *IL10* (24h), *EBI3* (24h), and *IFNB* (48h), whereas treatment with 60 μg triggered enhanced expression of several cytokines: *IL1* (24h), *IL6* (24h), *CXCL8* (24h), *IL10* (24h, 48h), *IL12B* (24h), *EBI3* (24h), and *IFNB* (24h) (**Figure 6**). These milk-derived nanosized structures slightly modulated the cytokine expression on moM1, with only a small increase observed in the expression of *CXCL8* (0.6 μg 48h), *IL10* (0.6 μg 24h), *IL12A* (60 μg 24h), *IL12B* (0.6 μg 24h), and *TNF* (0.6 μg 24h and 48h, 0.6 μg 24h) (**Figure 7**).

### 3.4 Release of key immune cytokines by porcine moMФ and moM1 after exposure to milk EVs

**Figure 8:**
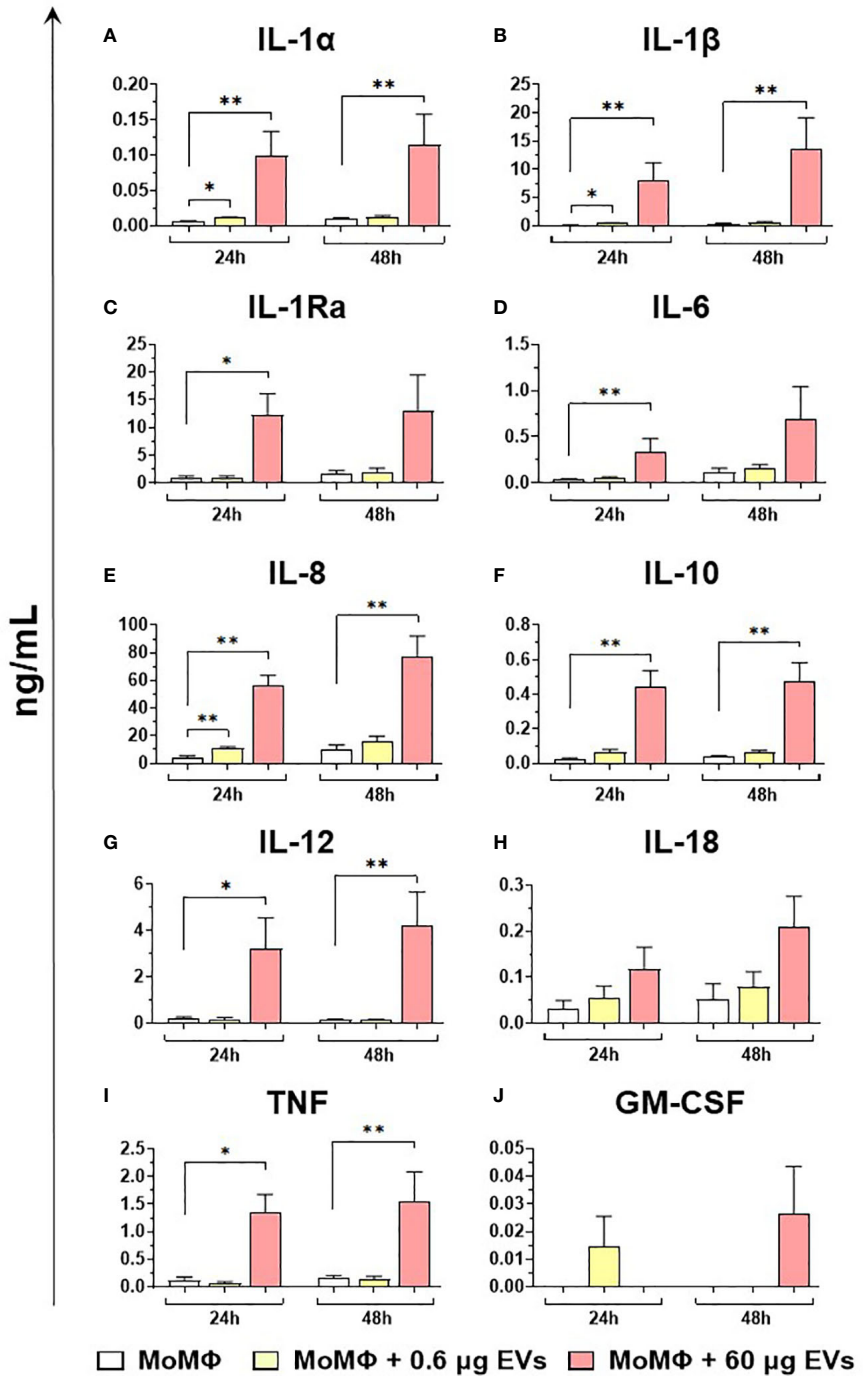
Release of key cytokines by moMФ after exposure to different concentrations of goat mEVs. Porcine moMФ were left untreated or treated with 0.6 or 60 μg of goat mEVs. At 24 and 48 h post-stimulation, culture supernatants were collected and levels of ten cytokines (IL-1α, IL-1β, IL-1Ra, IL-6, IL-8, IL-10, IL-12, IL-18, TNF, GM-CSF) **(A–J)** were determined using multiplex ELISA. Mean data and SD from three independent experiments are displayed. At 24 and 48 h post-treatment, values of mEVs-treated moMΦ were compared to the corresponding untreated control (moMΦ), using an unpaired T test of a Mann-Whitney test; **p < 0.01, *p < 0.05.

**Figure 9:**
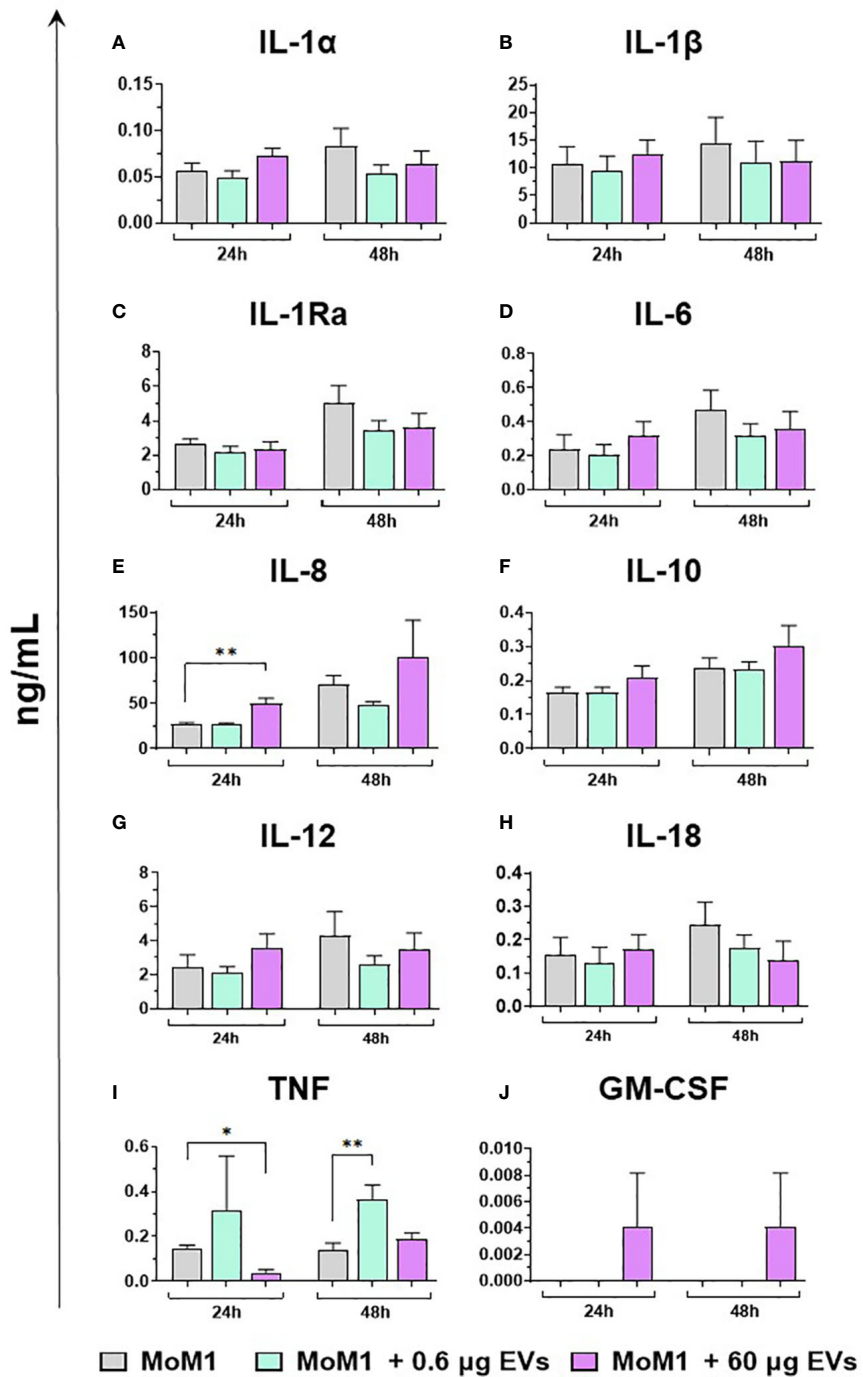
Release of key cytokines by moM1 after exposure to different concentrations of goat mEVs. Porcine moM1 were left untreated or treated with different concentrations of mEVs: 0.6 or 60 μg. At 24 and 48 h post-stimulation, culture supernatants were collected and levels of ten cytokines (IL-1α, IL-1β, IL-1Ra, IL-6, IL-8, IL-10, IL-12, IL-18, TNF, GM-CSF) **(A–J)** were determined using multiplex ELISA. Mean data and SD from three independent experiments are displayed. At 24 and 48 h post-treatment, values of mEVs-treated moM1 were compared to the corresponding untreated control (moM1), using an unpaired T test of a Mann-Whitney test; **p < 0.01, *p < 0.05.

Finally, the ability of mEVs to stimulate the release by macrophage subsets of the ten key cytokines already tested for gene expression was evaluated through ELISA multiplex. In detail, moMФ and moM1 were exposed to the same two doses of goat mEVs (0.6 or 60 μg), alongside untreated controls, and cytokines levels in culture supernatants were quantified at 24 and 48 h post-treatment. Exposure to the lowest dose (0.6 μg) did not affect cytokine contents in culture supernatants, with the exception of a small (but statistically significant) enhancement of IL-1α, IL-1β, and IL-8 levels (**Figure 8**). On the contrary, the exposition to 60 μg of goat mEVs promoted the release of several cytokines by moMФ, with statistical significance at 24 h (IL-1α, IL-1β, IL-1Ra, IL-6, IL-8, IL-10, IL-12, and TNF) and 48 h (IL-1α, IL-1β, IL-8, IL-10, IL-12, and TNF) post-treatment (**Figure 8**). For IL-18, we observed an increased release in culture supernatants of moMФ treated with 60 μg of goat mEVs, although without statistical significance (**Figure 8**). As expected, basal culture levels of pro-inflammatory cytokines (IL-1α, IL-1β, IL-6, IL-8, and IL-12), as well as IL-10 and TNF, were higher in moM1 compared to moMФ (**Figure S4**). Interestingly, the exposure to mEVs did not alter cytokine content in culture supernatants in a remarkable manner, with the exception of a raise for IL-8 in moM1 treated with 60 μg of goat mEVs (**Figure 9**). Levels of IFN-β in culture supernatants were also quantified using singleplex ELISA: we observed that goat mEVs did not trigger the release of type I IFN by either moMФ and moM1 (data not shown).

## 4 Discussion

Milk-derived extracellular vesicles (mEVs) have drawn researchers’ attention during the last few years for their potential applications in the biomedical field related to their considerable theranostic properties (1). Diverse studies described that mEVs can modulate immune cell response and inflammatory processes, likely improving inflammatory-based pathological conditions such as those of the gut (inflammatory bowel disease - IBD). For these characteristics, these natural nanocarriers might be used as adjuvant therapy or can be added to infant formulae when breast milk is not available, in order to prevent the development of necrotizing colitis (40, 41). Moreover, mEVs are one of the most promising delivery systems also in the EV field: they can be loaded with drugs and biologics, including nucleic acids, being an efficient shuttle toward target cells in particular for poorly adsorbable compounds (1, 42). They have been tested for the loading of several molecules as therapy for different pathologies, mainly cancer, with promising results in terms of efficacy and safety (43, 44). In cancer immunotherapy, numerous efforts have been made to improve the performance of EVs as drug delivery vehicles. Diverse techniques were implemented to load EVs with various cargo and several strategies were investigated to increase both their cellular uptake and their targeting ability, such as the use of cross-linkers (45). Before any potential biomedical application, it is crucial to investigate mEV impact on key immune cells, such as macrophages, which are at the frontline defense against both infective and not infective stressors (7). The impact of foreign molecules on these professional phagocytes may trigger unwanted immunotoxicity. In addition, macrophages have become an important therapeutic target in immunotherapy against several diseases, including cancers. In cancer immunotherapy, several macrophage-targeted strategies were developed, aiming at depleting tumor associated macrophages (TAM), inhibiting their recruitment, or reprogramming them toward an anti-tumor phenotype (10).

To date, very few studies investigated the biomedical application of goat mEVs. Some *in vitro* works were carried out in mice, where goat mEVs were labeled with commercial fluorophores to create nanoprobes (46–48). Experiments on a mouse macrophage cell line (RAW264.7) showed that these nanoprobes were efficiently internalized by macrophages, with a lack of cytotoxicity (47, 48). In the vivo experiment, goat mEVs as nanoplatforms were used to detect inflammatory processes: optical imaging confirmed the ability of goat mEV-nanoprobes to localize inflammatory processes, and flow cytometry of exudates from inflamed regions revealed that these molecules were efficiently up-taken by macrophage and neutrophil populations (47). In a glioblastoma xenograft model, a strong uptake of goat mEVs in tumor tissues was demonstrated, especially in tumor cells and tumor-associated macrophages (48). These studies confirmed the diagnostic capabilities of these nanoprobes, although researchers did not investigate in detail their effects on macrophages or other immune cells. Other studies were performed using mEVs of other species, such as bovine. Somiya and collaborators tested bovine mEVs on RAW264.7, showing no cytotoxic effect (49) and, more recently, their protective action on these cells against cisplatin-induced cytotoxicity was described (50). Nevertheless, other aspects beyond toxicity should be analyzed. After adsorption, EVs can transfer their molecular cargo into recipient cells (51), thus a detailed analysis of their immunomodulatory effects on receiving cells should be carried out.

In this work, we investigated the impact of goat mEVs on porcine macrophages in detail, analyzing both untreated (moMФ) and classically activated macrophages (moM1), the latter characterized by a pro-inflammatory antimicrobial phenotype (19). First, goat mEVs were isolated and characterized, then the best concentration to be administered was determined. We tested doses from 0.06 to 600 μg (protein weight) of goat mEVs and 24 h post-exposure a viability assay was employed to quantify cell viability. Only 600 μg amount presented cytotoxic effect on moMФ, thus we selected 60 μg for the immunological tests, in order to have the maximum effect with the smallest degree of toxicity. A lower quantity was also selected (0.6 μg), in order to evaluate whether the effects observed were dose-dependent. Goat mEVs were efficiently internalized by porcine macrophages, in agreement with previous studies on mEVs and human or murine macrophage cell lines (49–53), and primary human macrophages (54).

We observed that high doses of goat mEVs (60 μg) enhanced the expression of macrophage activation markers (MHC class I, MHC class II DR), and the induction/release of proinflammatory cytokines. We detected an increased expression/release of IL-1, IL-6, and TNF, which are indeed the most important pro-inflammatory cytokines of the innate immune response (55). IL-1α and IL-1β are interleukins produced and released at the early stages of infections, characterized by similar biological properties (56). IL-1α production is likely under the control of IL-1β (57), which is not only a potent pro-inflammatory cytokine, but also a key inducer of proliferation and differentiation of different CD4^+^ T cell subsets (58). Both IL-1, IL-6, and TNF trigger the release of several chemokines, such as IL-8, which in turn promotes the infiltration of leukocytes in the inflamed tissue (56). IL-1β, IL-6, and TNF are potent pyrogens, which enhance the synthesis of acute phase proteins (e.g. C reactive protein) from the liver (55). High doses of goat mEVs (60 μg) also enhanced the release of another member of the IL-1 superfamily: IL-18. IL-18 is a potent inducer of IFN-γ production (56) which can synergize with IL-12 to activate natural killer (NK) cells and cytotoxic T cells (59, 60). Even if the IL-18 increase was not statistically significant, our data showed that the gene expression of IL-12 subunits (*IL12A* and *IL12B*) was induced in moMФ following exposure to high doses of mEVs, in association with the increased release of IL-12. The concomitant release of both IL-12 and IL-18, alongside pro-inflammatory cytokines, suggests that goat mEVs can polarize macrophages toward an M1-like phenotype *in vivo*, with the subsequent enhancement of IFN-γ production and the activation of both NK cells and cytotoxic T cells. Nevertheless, we observed also the *EBI3* upregulation in moMФ after mEV treatment. EBI-3 is a subunit of IL-27 and IL-35 depending on the other subunit it combines with, p28 or p35 respectively (61). These two interleukins are part of the IL-12 family, where IL-27 is an immunoregulatory cytokine and IL-35 is a potent inhibitory cytokine (62). We might speculate that EBI-3 induction is a protective mechanism, put in place to avoid the development of an uncontrolled immune response.

Accordingly, we observed that porcine moMФ released two immune-suppressive cytokines (IL-1Ra and IL-10) in response to 60 μg of goat mEVs: IL-1Ra is a receptor antagonist, released to block further IL-1 activity (56), while IL-10 is a potent anti-inflammatory cytokine (63). These data are in line with a classical activation of porcine macrophages, which is characterized not only by the release of pro-inflammatory chemokines and cytokines but also by a significant IL-1Ra response and a small release of IL-10 (19).

Overall, these preliminary *in vitro* data suggest that these nanosized structures were able to polarize porcine macrophages towards an M1-like phenotype, which can be useful to enhance defense against intracellular pathogens and malignancies (63). Tumor progression is indeed associated with skewing and subversion of macrophage function toward a pro-tumor phenotype, with anti-inflammatory, pro-angiogenetic activities, that promote tumor growth and metastasis (64–66). A promising strategy in cancer immunotherapy is to reprogram TAM, shifting from a pro-tumor type to an anti-tumor state (M1 phenotype) (10, 66). The administration of mEVs loaded with therapeutic molecules might not only guarantee more efficient delivery of these molecules but might contribute to reprogramming tumor associated macrophages toward an M1-like tumoricidal phenotype. Regarding the effect of mEVs on cancer growth, a recent study reported that oral administration of bovine mEVs in mice after primary tumor resection determined a reduction of lung metastasis in breast cancer models (67), thus a positive effect of mEVs on key immune cells can be speculated.

Interestingly, we observed that these membrane-enclosed structures had a very weak effect on moM1, with modest induction of *RELA* gene expression, few pro-inflammatory cytokines (with no subsequent release), and a mild downregulation of some TLRs. The observed modest impact on moM1 represents a positive data for a clinical application of this molecules: when macrophages are already in a pro-inflammatory status (moM1), their activation seemed to be controlled, preventing potentially pathological over-response to stressors. Accordingly, we observed also the downregulation of *TLR4*, *TLR8*, and *TLR9* in porcine moMФ exposed to goat mEVs. The down-regulated expression of these PRRs may indeed represent a protective mechanism, put in place to tightly regulate the macrophage activation, to prevent the development of pathological inflammatory responses or even autoimmunity (68). Overall, these data are in favor of a clinical application of these nanosized delivery systems *in vivo*.

Indeed, our results indicate that goat mEVs polarized macrophages toward a pro-inflammatory and tumoricidal M1-like phenotype, but they did not have a significant impact when these cells were already classically activated (moM1). These preliminary *in vitro* data hint at a potential application of goat mEVs in the biomedical field. These molecules are indeed able to activate untreated macrophages, after being internalized, but then other mechanisms seem to occur in controlling this activation, preventing a potentially pathological over-response to stressors.

## Data availability statement

The raw data supporting the conclusions of this article will be made available by the authors, without undue reservation.

## Ethics statement

The animal study was reviewed and approved by Institutional Ethics Committee of the Istituto Zooprofilattico Sperimentale della Sardegna.

## Author contributions

GF, SM, KC, and ER conceived the study. GF, SM, CC, LM, FD, SZ, FF, LPao, TC, SD, AA, EC, and LPas conducted the experiments. GF, SM, AA, SD, EC, and LPas performed the formal analysis. GF, and SM wrote the first draft of the manuscript, which was reviewed and edited by KC, and ER. AO, KC, and ER provided resources and funding acquisition. All authors have read and agreed to the submitted version of the manuscript.
