# Corrigendum: Goat milk extracellular vesicles: immuno-modulation effects on porcine monocyte-derived macrophages *in vitro*

**DOI:** 10.3389/fimmu.2023.1247751

**Published:** 2023-07-03

**Authors:** Giulia Franzoni, Samanta Mecocci, Chiara Grazia De Ciucis, Lorena Mura, Filippo Dell’Anno, Susanna Zinellu, Floriana Fruscione, Livia De Paolis, Tania Carta, Antonio G. Anfossi, Silvia Dei Guidici, Elisabetta Chiaradia, Luisa Pascucci, Annalisa Oggiano, Katia Cappelli, Elisabetta Razzuoli

**Affiliations:** ^1^ Department of Animal Health, Istituto Zooprofilattico Sperimentale della Sardegna, Sassari, Italy; ^2^ Department of Veterinary Medicine, University of Perugia, Perugia, Italy; ^3^ National Reference Center of Veterinary and Comparative Oncology (CEROVEC), Istituto Zooprofilattico Sperimentale del Piemonte, Liguria e Valle d’Aosta, Genova, Italy; ^4^ Department of Biomedical Sciences, School of Medicine, University of Sassari, Sassari, Italy; ^5^ Department of Veterinary Medicine, University of Sassari, Sassari, Italy

**Keywords:** milk extracellular vesicles, pig, macrophages, classical activation, cytokines, toll-like receptors

In the published article, an author name was incorrectly written as “Katia Capelli”. The correct spelling is “Katia Cappelli”.

The authors apologize for this error and state that this does not change the scientific conclusions of the article in any way. The original article has been updated.

