# Supplementary material for: Goat milk extracellular vesicles: immuno-modulation effects on porcine monocyte-derived macrophages in vitro
Source: Front Immunol. 2023 Jun 16;14:1209898. doi: 10.3389/fimmu.2023.1209898 (PMC10352104; doi:10.3389/fimmu.2023.1209898)
Supplement: Supplementary file 1 [file DataSheet_1.pdf]

Supplementary Material

Goat milk Extracellular Vesicles: immuno-modulation effects on porcine monocyte-derived macrophages *in vitro*

Giulia Franzoni [1]*, Samanta Mecocci [2]*, Chiara Grazia De Ciucis [3], Lorena Mura [1,4], Filippo Dell'Anno [3], Susanna Zinellu [1], Floriana Fruscione [3], Livia De Paolis [3], Carta Tania [1,5], Antonio G. Anfossi [5], Silvia Dei Giudici [1], Elisabetta Chiaradia [2], Luisa Pascucci [2], Annalisa Oggiano [1], Katia Capelli [2#], Elisabetta Razzuoli [3#]

*** Correspondence:** Giulia Franzoni: giulia.franzoni@izs-sardegna.it; Samanta Mecocci: samanta.mecocci@unipg.it

Table S1. Oligonucleotide Primer and Probe sets for detection of several porcine viruses in pig blood.

Virus	Sequences	Reference
PCV2	F: 5'-TGGCCCGCAGTATTCTGATT-3'<br>R: 5'-CAGCTGGGACAGCAGTTGAG-3'<br>Probe 5'-FAM-CCAGCAATCAGACCCCGTTGGAATG-BHQ1-3'	Opriessing et al., 2003
PPV	F: 5'-GAAGACTGGATGATGACAGATCCA-3'<br>R: 5'-TGCTGTTTTTGTTCTTGCTAGAGTAA-3'<br>Probe 5'-VIC-AATGATGGCTCAAACCGGAGGAGA-BHQ1-3'	Song et al., 2010
ASFV	F: 5'-CTGCTCATGGTATCAATCTTATCG A-3'<br>R: 5'-GATACCACAAGATCRGCCGT-3'<br>Probe 5'-FAM-CCACGGGAGGAATACCAACCCAGTG-TAMRA-3'	King et al., 2003

Table S2. Oligonucleotide sequences of primer pairs used for RT-qPCR in pig moMΦ and moM1.

Gene	Sequences	Amplicon length	Reference / Accession
IL1B	F: 5'-AATTCGAGTCTGCCCTGTACCC-3'<br>R: 5′-TGGTGAAGTCGGTTATATCTTGGC-3′	111	Razzuoli et al. 2017
IL6	F: 5'-CAGAGATTTTGCCGAGGATG-3'<br>R: 5'-TGGCTACTGCCTTCCCTACC-3'	132	Razzuoli et al. 2017
IL10	F: 5′-AGCCAGCATTAAGTCTGAGAA-3<br>R: 5′-CCTCTCTTGGAGCTTGCTAA-3′	394	Carta et al. 2021
IL12A	F: 5′-ATGCCTCAACCACTCCCAAA-3′<br>R: 5′-TGTGCTGGTTTTATCTTTGGTGA-3′	136	Mecocci et al., 2022
IL12B	F: 5'-TCAGGGACATCATCAAACCA-3'<br>R: 5'-GAACACCAAACATCAGGGAAA-3'	141	Carta et al. 2021
TNFA	F: 5'-TGCCTACTGCACTTCGAGGTTATC-3'<br>R: 5'-GTGGGCGACGGGCTTATCTG -3'	126	Razzuoli et al. 2013

CXCL8	F: 5’-TTCGATGCCAGTGCATAAATA-3’<br>R: 5’-CTGTACAACCTTCTGCACCCA-3’	175	Razzuoli et al. 2017
IL18	F: 5′-CGTGTTTGAGGATATGCCTGATT-3<br>R: 5′-TGGTTACTGCCAGACCTCTAGTGA-3′	106	Razzuoli et al. 2017
EBI3	F: 5’-CAACGTCACAGCCATCCAC-3’<br>R: 5’-GGTTTCCACTGCACCCAA-3’	141	Mecocci et al., 2022
IFNB	F: 5’-AGTTGCCTGGGACTCCTCAA-3’<br>R: 5’-CCTCAGGGACCTCGAAGTTCAT-3’	60	Razzuoli et al. 2011
DEFB1	F: 5’CTGTTAGCTGCTTAAGGAATAAAGGC-3’<br>R: 5’-TGCCACAGGTGCCGATCT-3’	81	Razzuoli et al. 2017
RELA	F: 5’-CGAGAGGAGCACGGATACCA-3’<br>R: 5’-GCCCCGTGTAGCCATTGA-3’	62	Razzuoli et al. 2017
TLR1	F: 5’-AGATTTCGTGCCACCCTATG-3’<br>R: 5’-CCTGGGGGATAAACAATGTG-3’	277	Franzoni et al. 2021
TLR2	F: 5’-CGGCTTCCAAGGATGGAGAAA-3’<br>R: 5’-TCCAGAGAGTTGACCTTGCAG-3’	72	Franzoni et al. 2022c
TLR3	F: 5’-TGAAGAACTTGATTTCCTTGGCA-3’<br>R: 5’- GGCATGAAAACACCCTGGAG-3’	94	Franzoni et al. 2021
TLR4	F: 5'-TGGCAGTTTCTGAGGAGTCATG-3'<br>R: 5'-CCGCAGCAGGGACTTCTC-3’	72	Razzuoli et al. 2017
TLR5	F: 5’-TCAAAGATCCTGACCATCACA-3’<br>R: 5’-CCAGCTGTATCAGGGAGCTT-3’	60	Razzuoli et al. 2017
TLR7	F: 5’-GTGGAAATTGCCCTCGTTGT-3’<br>R: 5’-GATGGATCTGTAGGGGAGCA-3’	78	Franzoni et al. 2021
TLR8	F: 5’-AAGACAACCAGTTACGTGAAATACC-3’<br>R: 5’-GGGTGTTAAAAGATAATGACAGCAC-3’	237	Yoo et al. 2019
TLR9	F: 5’-AGGACTTCATGCCAAACTGC-3’<br>R: 5’-CGAGCAAACATCTCCGACTG-3’	91	Franzoni et al. 2021
GAPDH	F: 5’-ACCCAGAAGACTGTGGATGG-3’<br>R: 5’-ACGCCTGCTTCACCACCTTC-3’	247	Mecocci et al., 2022

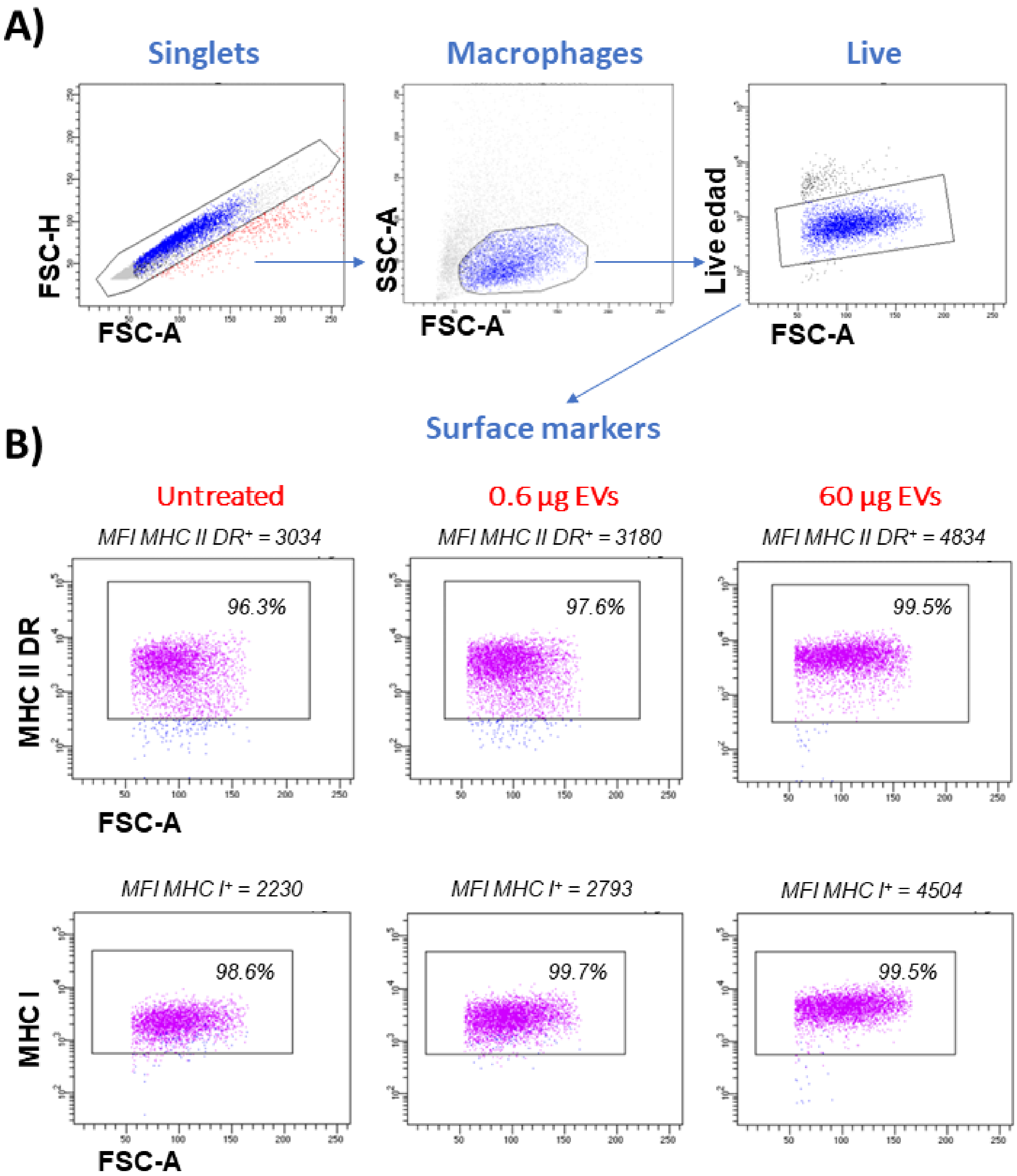

Figure S1. Gating strategy to determine surface expression of MHC II DR DR and MHC I on porcine macrophages. Porcine macrophages were left untreated of stimulated with goat milk EVs. 24 and 48 h post-stimulation, dimension (FSC-A) and surface expression of MHC II DR and MHC I were investigated using flow cytometry. In panel a, gating strategy used to investigate surface marker expression is presented: exclusion of doublets, gating on viable moMΦ, then assessing the staining for surface markers. In panel b, representative dot plots of surface marker screened (MHC II DR and MHC I) are displayed.

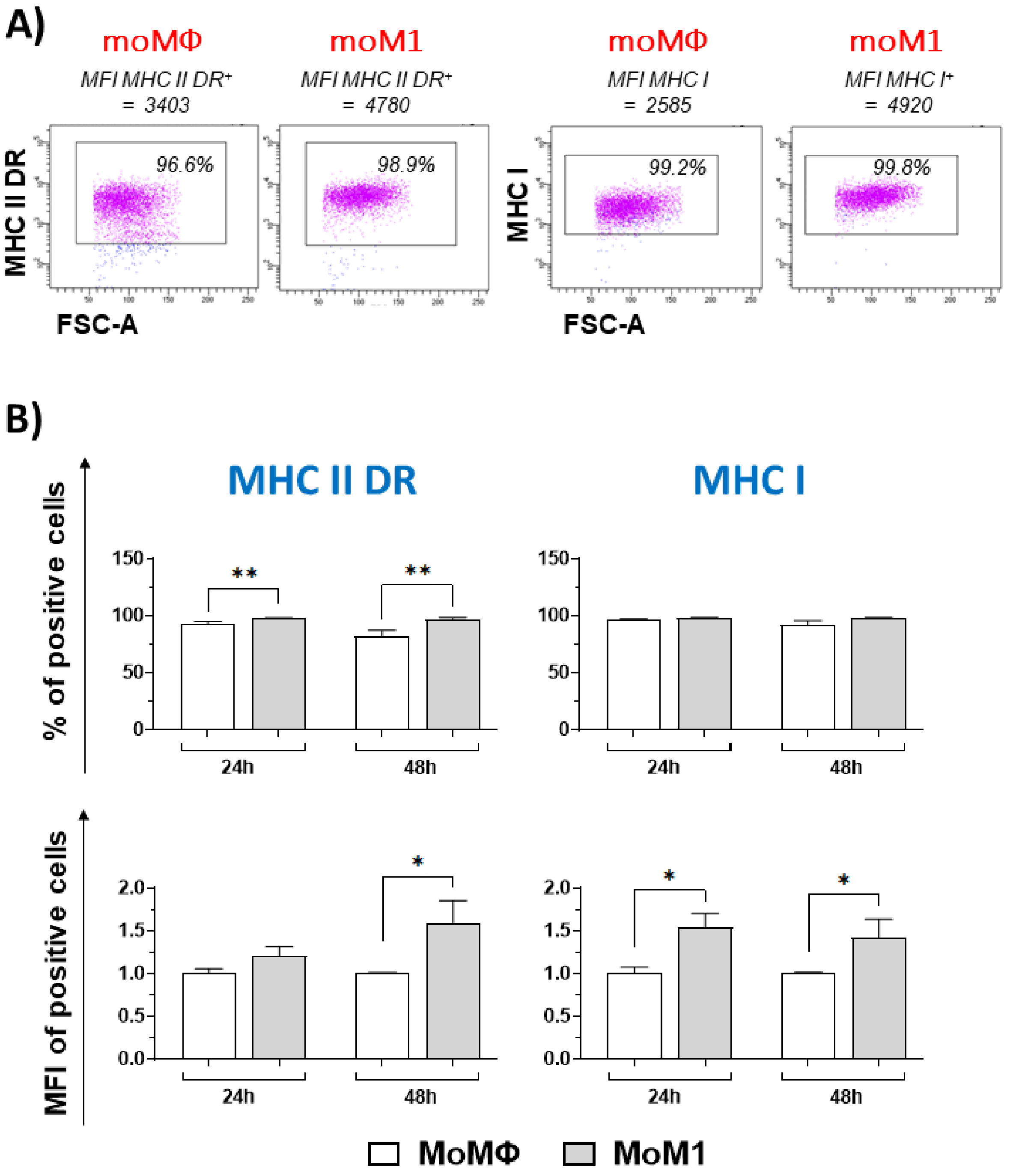

Figure S2. Surface expression of MHC II DR and MHC I on porcine macrophage subsets. Porcine moMΦ were left untreated or stimulated with IFN-γ and LPS (both at 100 ng/mL) to achieve classical activation (moM1). 24 and 48 h post-stimulation, surface expression of MHC II DR and MHC I were investigated using flow cytometry. In panel A, representative dot plots are displayed. In panel B, percentage of positive cells and mean fluorescent intensity (MFI) of positive cells are presented. MFI data are expressed as fold change relative to the mock-infected un-activated condition (moMΦ). Mean data and SD from four independent experiments using different blood donor pigs are displayed. Values of macrophage subsets (moMΦ and moM1) were compared using an unpaired T test of a Mann-Whitney test; ** $p < 0.01$, * $p < 0.05$.

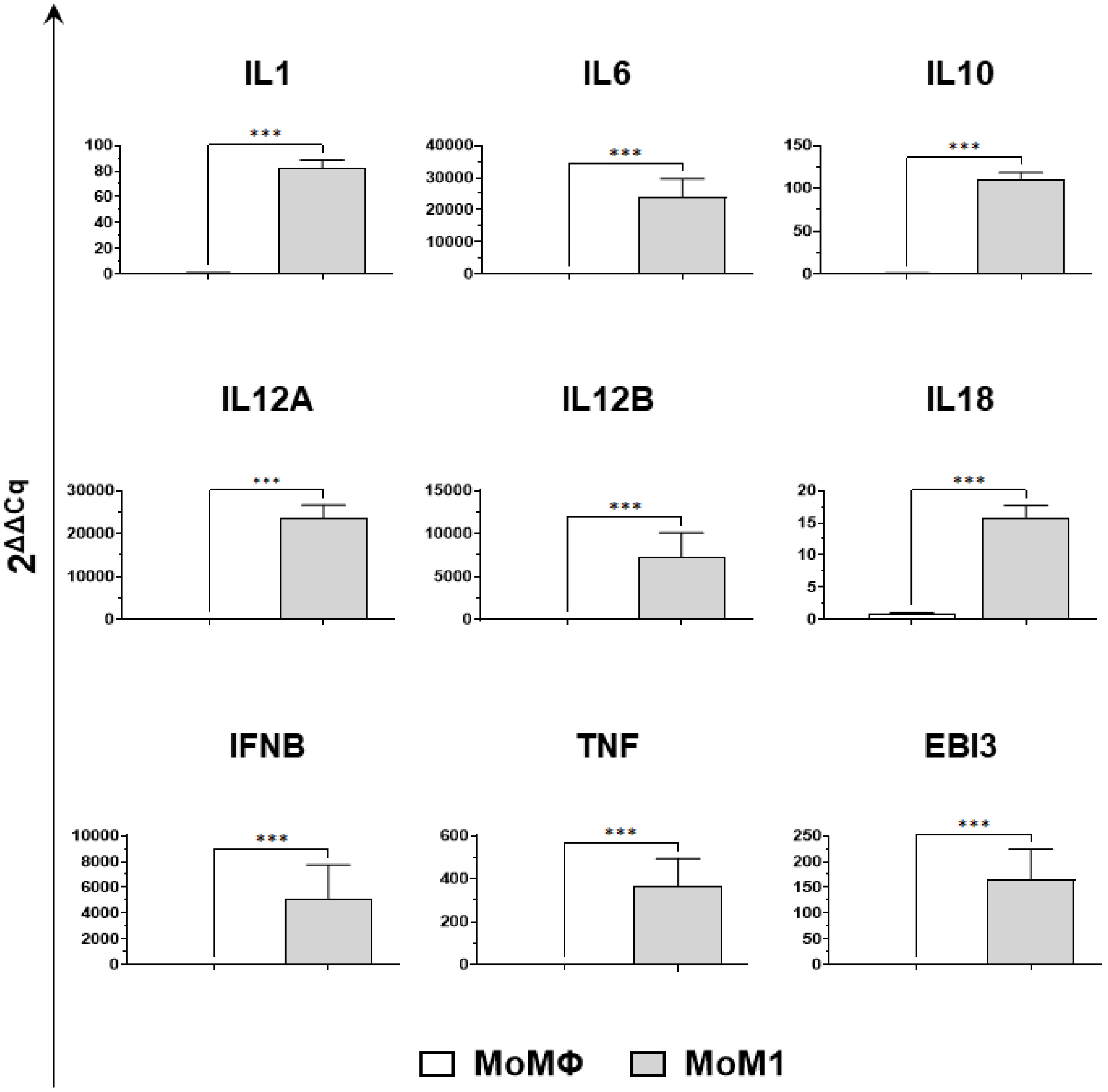

Figure S3. Expression of key cytokine genes in porcine macrophage subsets. Porcine moMΦ were left untreated or stimulated with IFN-γ and LPS (both at 100 ng/mL) to achieve classical activation (moM1). 24h post-stimulation, gene expression levels of were determined using RT-qPCR. Data were normalized on the values of untreated control group (moMΦ) and expressed as $2^{-\Delta\Delta Cq}$. Mean data and SD from four independent experiments using different blood donor pigs are displayed. Values of treated macrophages were compared to the untreated control (moMΦ), using an unpaired T test of a Mann-Whitney test; *** $p < 0.001$.

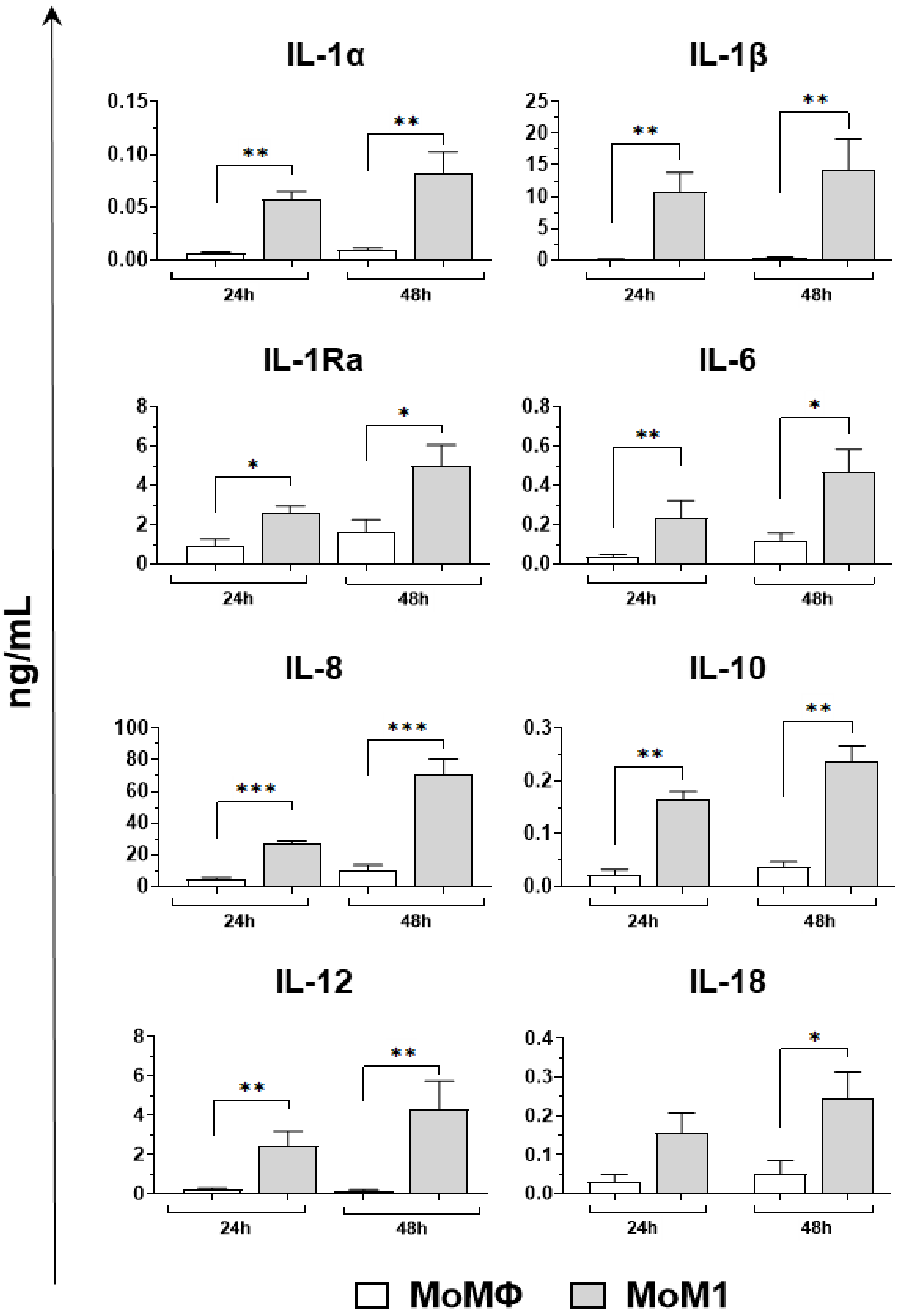

Figure S4. Levels of key cytokines in culture supernatants on porcine macrophage subsets. Porcine moMΦ were left untreated or stimulated with IFN-γ and LPS (both at 100 ng/mL) to achieve classical activation (moM1). After 24 and 48 h, levels of IL-1α, IL-1β, IL-1Ra, IL-6, IL-8, IL-10, IL-12, IL-18 in culture supernatants were investigated using multiplex ELISA. Mean data and SD from three independent experiments using different blood donor pigs are displayed. Values of macrophage subsets (moMΦ and moM1) were compared using an unpaired T test of a Mann-Whitney test; ** $p < 0.01$, * $p < 0.05$.

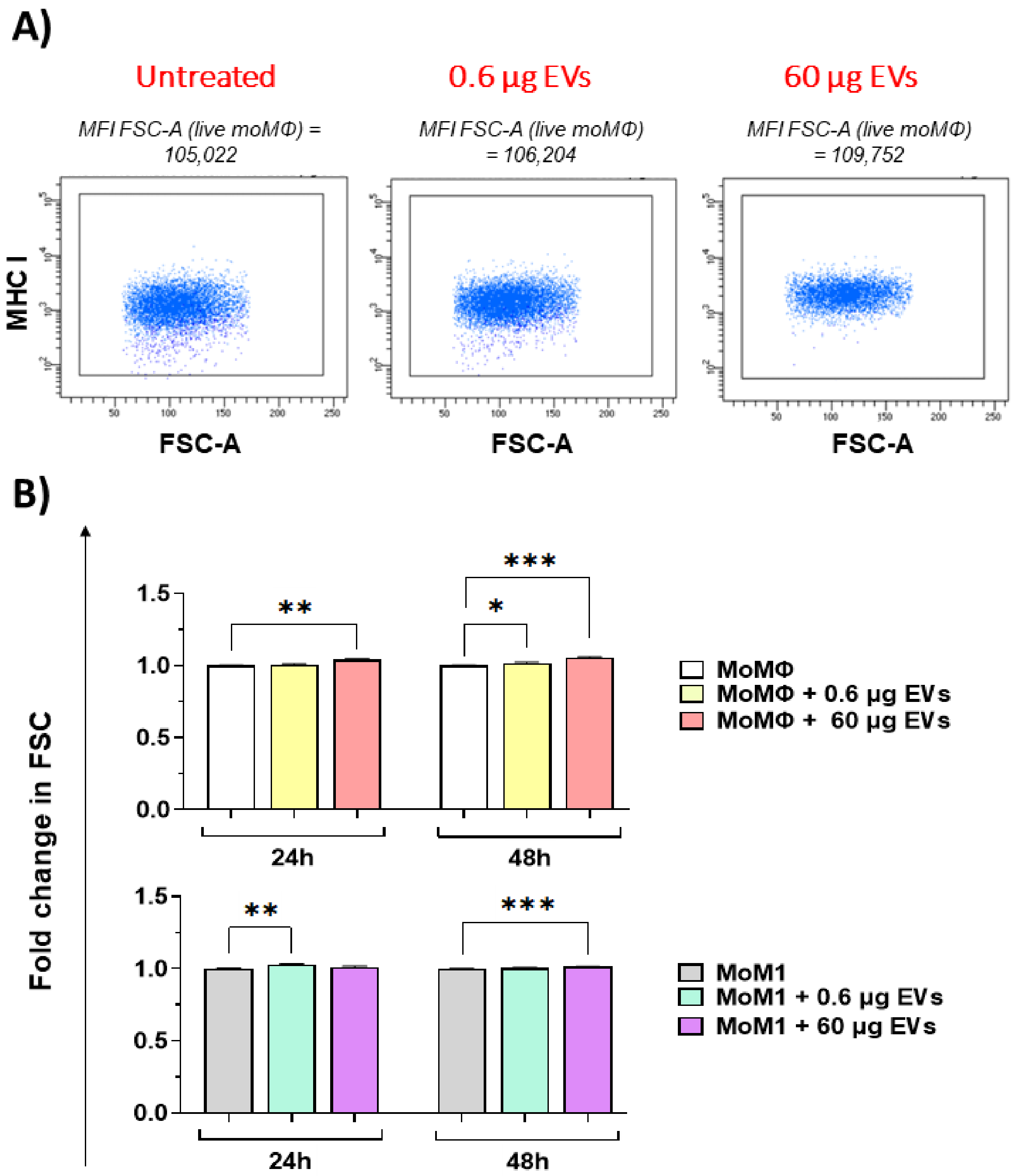

Figure S5. Goat milk EVs effect on porcine moMΦ or moM1 size. Porcine moMΦ or moM1 were left untreated or treated with different doses of EVs (0.6 or 60 µg). 24 and 48 h post-stimulation dimension was evaluated using flow cytometry, by quantification of forward scatter area (FSC-A, geometric mean). In panel A, representative dot plots of moMΦ un-treated or exposed to diverse doses of EVs are displayed. In panel B, mean data for quadruplicate biological replicates and standard deviation (SD) are presented. FSC data are presented as fold-change relative to the corresponding untreated condition (moMΦ or moM1). Values of EVs-treated macrophages were compared to the untreated control (moMΦ or moM1), using an unpaired T test of a Mann-Whitney test. *** $p < 0.001$, ** $p < 0.01$, * $p < 0.05$.
